# Association between red blood cell distribution width to albumin ratio and pneumonia risk in acute ischemic stroke: evidence from two independent cohorts

**DOI:** 10.3389/fnut.2026.1847555

**Published:** 2026-08-17

**Authors:** Jin Wang, Yaxin Zhang, Yadong Wu, Shulan Lin, Fan Wu, Shuqi Lin, Min Zhang, Weihong Zheng

**Affiliations:** Department of Neurology, Xiamen Humanity Hospital, Fujian Medical University, Xiamen, Fujian, China

**Keywords:** acute ischemic stroke, albumin, intensive care unit, pneumonia, red blood cell distribution width

## Abstract

**Background:**

Pneumonia is a common and severe complication in acute ischemic stroke (AIS) patients, particularly those requiring intensive care. Early prediction of pneumonia risk can enable timely interventions and improve clinical outcomes. The Red Blood Cell Distribution Width to Albumin Ratio (RAR), combining markers of inflammation (RDW) and nutritional status (albumin), has potential as a predictor of pneumonia risk in AIS patients.

**Methods:**

This retrospective cohort study included AIS patients from MIMIC-IV (*n* = 1,266) and XMHH (*n* = 294). Pneumonia incidence was compared across tertiles of RAR. The relationship between RAR and pneumonia was assessed using multivariable logistic regression. Restricted cubic splines (RCS) were used to explore the non-linear relationship between RAR and pneumonia risk. Predictive performance was evaluated using receiver operating characteristic (ROC) curves and area under the curve (AUC). *E*-value analysis was performed to assess the robustness of the association and the impact of unmeasured confounders.

**Results:**

Elevated RAR was significantly associated with an increased risk of pneumonia in both cohorts. In the MIMIC-IV cohort, the incidence of pneumonia was higher in the highest RAR group (T3) compared to the lowest (T1) (21.75% vs. 7.11%, *p* < 0.001). In fully adjusted model, the OR for pneumonia in the highest RAR group was 2.11 (1.30–3.41). A similar trend was observed in the XMHH cohort, with an OR of 3.94 (1.27–12.27). Exploratory subgroup analyses suggested that the association between RAR and pneumonia appeared more pronounced among patients without atrial fibrillation and without renal disease in the MIMIC-IV cohort; however, these findings were not replicated in the XMHH cohort and should be interpreted cautiously.

**Conclusion:**

RAR was independently associated with pneumonia during ICU stay among patients with AIS. As a readily available and low-cost biomarker, RAR may serve as an adjunctive marker for early risk stratification. However, its discriminatory performance was modest, and its clinical value beyond established predictors remains uncertain. Prospective studies are needed to confirm these findings.

## 1 Introduction

Acute ischemic stroke (AIS) is one of the leading causes of death and long-term disability worldwide, with a growing burden on both individuals and healthcare systems (1, 2). Among the many complications that arise post-stroke, pneumonia remains the most prevalent and consequential, significantly increasing mortality rates, prolonging hospital stays, and delaying recovery (3, 4). Despite significant advances in acute stroke management, the incidence of pneumonia in stroke patients, particularly those requiring intensive care, remains high (5, 6). Patients with AIS requiring ICU admission generally have more severe neurological or systemic illness and are more frequently exposed to pneumonia-related factors, such as impaired consciousness, dysphagia, aspiration, invasive procedures, and mechanical ventilation. Therefore, ICU patients constitute a clinically important high-risk population in whom the assessment of readily available biomarkers may be particularly relevant. Early identification of those at increased risk for pneumonia could improve patient outcomes by enabling timely preventive measures. However, reliable biomarkers for early detection are still lacking (7).

Red blood cell distribution width (RDW) is a widely available, inexpensive marker that has shown promise in predicting outcomes across a variety of diseases, including cardiovascular diseases, infections, and inflammatory conditions (8). RDW reflects the degree of variation in red blood cell size, a process closely linked to systemic inflammation, oxidative stress, and endothelial dysfunction—pathophysiological processes that are critical in both stroke and pneumonia development (9). In recent years, elevated RDW levels have been associated with worse outcomes in stroke patients (10), but its direct relationship with pneumonia in this population remains largely unexplored.

Albumin (Alb), an essential plasma protein, is a well-established marker of nutritional status and inflammation (11). Lower albumin levels indicate systemic inflammation and poor nutritional status, both of which increase susceptibility to infections (12). In critically ill patients, including those with stroke, albumin has been used to predict complications like pneumonia, yet its role as an isolated predictor remains limited (13). When combined with RDW, red blood cell distribution width to albumin ratio (RAR) has emerged as a promising biomarker, reflecting both inflammatory burden and nutritional deficiency, two key factors contributing to the development of infections like pneumonia. RAR has been evaluated in several stroke-related settings. Previous studies have reported associations between RAR and stroke severity, functional prognosis, and short-term mortality in patients with acute ischemic stroke, suggesting that RAR may reflect systemic inflammation, nutritional status, and overall disease burden in this population (14). In parallel, several albumin-based or inflammation-related composite biomarkers, such as the fibrinogen-to-albumin ratio, lactate dehydrogenase-to-albumin ratio, albumin-to-globulin ratio, and geriatric nutritional risk index, have been investigated for their associations with stroke-associated pneumonia (15–18). These studies indicate that inflammatory and nutritional imbalance may contribute to pneumonia susceptibility after stroke. Several clinical models have been developed for stroke-associated pneumonia assessment, including A^2^DS^2^, AIS-APS, and ISAN. These models combine established predictors such as age, stroke severity, dysphagia, prestroke dependence, consciousness level, and comorbidities, and have demonstrated useful discriminatory performance in external validation studies (19–21). In parallel, recent studies have investigated circulating inflammatory and nutritional biomarkers, including procalcitonin, C-reactive protein, interleukin-6, serum amyloid A, and albumin-based composite indices (7, 22, 23). However, biomarkers generally provide only modest discrimination when used alone, and their incremental value beyond established clinical models remains uncertain. Within this context, RAR may represent a readily available composite indicator of inflammatory and nutritional status, but it should be evaluated as an adjunct rather than an alternative to established clinical assessment tools. However, evidence regarding the association between RAR and pneumonia risk in ICU patients with acute ischemic stroke remains limited. Therefore, the incremental contribution of the present study is to evaluate whether RAR is associated with pneumonia risk in this high-risk ICU population and to validate this association using two independent cohorts.

This study aims to address this gap by investigating the association between RAR and the risk of pneumonia in AIS patients. We hypothesize that higher RAR values will correlate with an increased risk of pneumonia and that RAR could serve as an easily accessible, cost-effective predictive tool for early identification of high-risk patients. By providing a comprehensive evaluation of RAR, we hope to establish a simple yet effective tool for predicting pneumonia risk in AIS patients, leading to earlier interventions and better outcomes.

## 2 Materials and methods

### 2.1 Study design and data sources

This retrospective cohort study utilized two independent cohorts: the MIMIC-IV (Medical Information Mart for Intensive Care) database and the XMHH (Xiamen Humanity Hospital) cohort. The MIMIC-IV database is a publicly available dataset containing de-identified clinical data from adult ICU patients at Beth Israel Deaconess Medical Center, Boston, USA (24). The dataset includes a wide range of variables, including demographic information, vital signs, laboratory results, medications, and clinical outcomes. The XMHH cohort consists of data collected from patients admitted to the ICU at Xiamen Humanity Hospital, between 2024 and 2025.

For the MIMIC-IV cohort, the study was conducted using publicly available, de-identified data. The use of this database was approved by the Institutional Review Board (IRB) of Beth Israel Deaconess Medical Center, and informed consent was waived due to the retrospective, de-identified nature of the data. The XMHH cohort was obtained in compliance with the respective Institutional Review Board (IRB) guidelines, with approval from Xiamen Humanity Hospital (IRB approval number: HAXM-MEC-20260312-012-01), and patient consent was waived due to the retrospective nature of the study.

### 2.2 Population selection criteria

**Figure 1:**
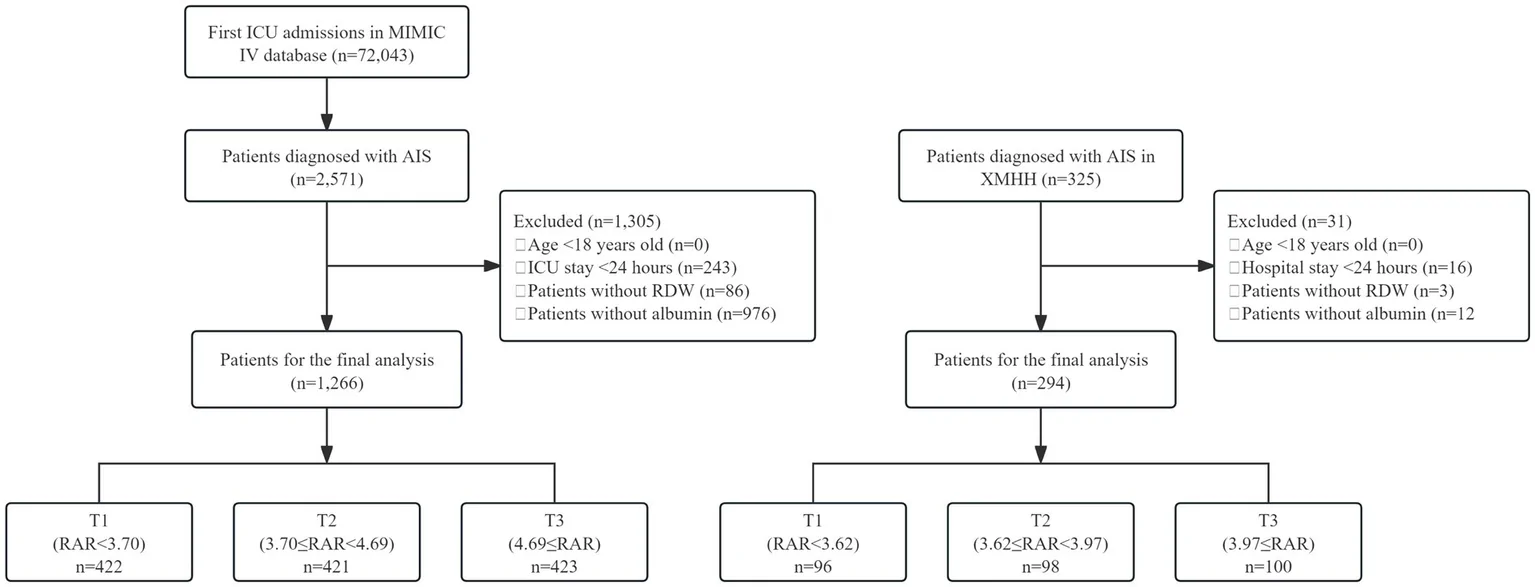
Flowchart of patient selection.

We included adult patients (aged ≥18 years) diagnosed with AIS and admitted to the ICU. The selection process is outlined in Figure 1. In the MIMIC-IV cohort, 72,043 patients were initially admitted to the ICU, of whom 2,571 were diagnosed with AIS. After applying the exclusion criteria, 1,305 patients were excluded due to reasons such as age <18 years (0 patients), ICU stay <24 h (243 patients), missing RDW data (86 patients), and missing albumin data (976 patients). This left a final cohort of 1,266 AIS patients from MIMIC-IV. In the XMHH cohort, 325 patients with AIS were initially considered, with exclusions for age <18 years (0 patients), ICU stay <24 h (16 patients), missing RDW data (3 patients), and missing albumin data (12 patients), resulting in 294 patients included for analysis. Patients were grouped into three categories based on their RAR levels.

### 2.3 Data extraction

Data were extracted from both the MIMIC-IV and XMHH cohorts. Key demographic, clinical, laboratory, and treatment-related data were collected. Demographic variables included age, sex, and race. Clinical comorbidities, including hypertension, diabetes, atrial fibrillation, myocardial infarction, chronic pulmonary disease, and renal disease, were recorded.

Vital signs, including heart rate, mean blood pressure (MBP), respiratory rate, and Glasgow Coma Scale (GCS) scores, were obtained from clinical records within the first 24 h of ICU admission. Laboratory results, including red blood cell distribution width (RDW), albumin, white blood cell count (WBC), creatinine, blood urea nitrogen (BUN), alanine aminotransferase (ALT), aspartate aminotransferase (AST), glucose, prothrombin time (PT), and international normalized ratio (INR), were also recorded within the first 24 h of ICU admission. Peripheral blood samples obtained within the first 24 h of ICU admission were used to capture the early systemic response while minimizing confounding from subsequent clinical interventions, such as medications, nutritional support, or prolonged ICU management. This time window was selected because previous studies have shown that peripheral inflammatory markers, including C-reactive protein, white blood cell count, and interleukin-6, change substantially during the first 24 h after acute stroke, reflecting the early inflammatory response to the disease process (25, 26). In addition, early blood biomarkers have been reported to be associated with stroke-associated pneumonia, supporting the clinical relevance of early laboratory assessment in this population (23). The RAR was calculated as RDW (%) divided by serum albumin concentration (g/dL), using the first available measurements within this period. Although serial measurements may better characterize the dynamic changes of RDW, albumin, and RAR, multi-timepoint monitoring was not performed in the present retrospective study because subsequent laboratory testing was not standardized. Prior studies have suggested that RDW measured at different time points after acute ischemic stroke may provide additional prognostic information, and this should be further evaluated in future prospective studies (27). Additionally, treatment data were collected, including whether patients received mechanical thrombectomy, recombinant tissue plasminogen activator (rtPA), antiplatelet therapy, and anticoagulation therapy. These treatment-related factors were incorporated into the analysis to assess their influence on the development of pneumonia.

For missing data, variables with less than 30% missingness were handled using multiple imputation by chained equations (MICE) with the random forest method, and five imputed datasets were generated. The primary analysis was initially performed using the first completed dataset. As a sensitivity analysis, regression models were repeated across all five imputed datasets, and the estimates were pooled according to Rubin’s rules. A complete-case analysis using the same model specifications was also performed. The missing data patterns are displayed in Supplementary Figure S1.

### 2.4 Outcomes

The primary outcome was pneumonia documented during the ICU stay. In the MIMIC-IV cohort, pneumonia was identified using diagnosis codes recorded during hospitalization. Because the timing of pneumonia onset relative to hospital admission, ICU admission, and RAR measurement could not be consistently determined, pneumonia present on admission could not be reliably excluded. In the XMHH cohort, pneumonia was identified according to the clinical diagnosis documented in the medical records, and patients with pneumonia present before ICU admission were excluded through medical record review. Thus, the XMHH outcome more closely represented newly diagnosed pneumonia during the ICU stay, whereas the MIMIC-IV outcome represented pneumonia documented during hospitalization regardless of the precise onset time.

### 2.5 Statistical analysis

Statistical analyses were performed using R (version 4.4.2). Descriptive statistics were used to summarize the baseline characteristics of the study populations. Continuous variables were expressed as means with standard deviations or medians with interquartile ranges, depending on their distribution. Categorical variables were presented as counts and percentages. Differences between groups were evaluated using the chi-square test for categorical variables and the Mann–Whitney U test for continuous variables.

For survival analysis, Kaplan–Meier survival curves were constructed to evaluate the 30-day and 90-day survival rates between patients with and without pneumonia. Log-rank tests were used to assess differences in survival distributions between groups. ROC curves were generated to evaluate the discriminatory performance of RAR, GCS, RDW, and albumin. AUCs with 95% confidence intervals were calculated, and pairwise differences between AUCs were assessed using the DeLong test. Logistic regression was employed to assess the association between RAR and pneumonia risk. Multivariable logistic regression models were constructed to adjust for potential confounders. Model 1 was unadjusted. Model 2 was adjusted for age, sex, heart rate, MBP, and respiratory rate. Model 3 was further adjusted for WBC, GCS score, creatinine, BUN, ALT, AST, glucose, and use of rtPA. Potential confounders were selected based on clinical relevance, previous literature, and univariable analyses. Multicollinearity was assessed using variance inflation factors, and variables with a VIF > 5 were not included simultaneously in the multivariable model. Odds ratios (ORs) with 95% confidence intervals (CIs) were calculated to estimate the association between RAR and pneumonia. Additionally, we used Restricted cubic spline (RCS) regression to model the non-linear relationship between RAR and pneumonia risk. This analysis was adjusted for the same potential confounders, and we tested for non-linearity using likelihood ratio tests. Subgroup analyses were conducted to examine the association between RAR and pneumonia in different subgroups defined by age, sex, and comorbid conditions. Logistic regression was used to calculate the odds ratios (OR) and 95% confidence intervals (CIs) in these subgroups. Finally, we calculated *E*-values to assess the potential impact of unmeasured confounders. The *E*-value indicates the minimum strength of an unmeasured confounder that could fully explain away the observed association between RAR and pneumonia. An exploratory nomogram incorporating age, RAR, heart rate, respiratory rate, WBC, BUN, and ALT was developed in the MIMIC-IV cohort. Its discrimination, calibration, and clinical utility were evaluated using the AUC, a 500-resample bootstrap-corrected calibration curve, and decision curve analysis, respectively.

## 3 Results

### 3.1 Baseline characteristics of the study population

In total, 1,266 AIS patients from the MIMIC-IV cohort and 294 from the XMHH cohort were included. Baseline characteristics differed between the MIMIC-IV and XMHH cohorts (Supplementary Table S1). MIMIC-IV patients were generally older, had greater comorbidity burden, lower albumin levels, and higher RAR values, with notable differences in acute stroke treatments. However, pneumonia incidence was similar between cohorts (13.82% vs. 13.61%, *p* = 0.922). Patients were grouped into three tertiles based on the RAR (Table 1). Higher RAR values were associated with older age, a higher prevalence of comorbidities (e.g., diabetes, AF, chronic pulmonary disease, and renal disease), and worse vital signs at admission (e.g., higher heart rate, lower MBP, and higher respiratory rate). Laboratory results indicated higher WBC, lower red blood cell counts, and lower albumin levels in higher RAR groups. The incidence of pneumonia was significantly higher in the T3 group (21.75%) compared to T1 (7.11%) (*p* < 0.001). The use of rtPA was also significantly higher in the T3 group (10.64%) compared to T1 (4.98%) (p < 0.001), while the proportion of patients receiving mechanical thrombectomy was similar across groups (*p* = 0.068). Anticoagulation therapy was used in most patients, with no significant differences between RAR groups (*p* = 0.087). Antithrombotic therapy was defined as the documented use of antiplatelet or anticoagulation therapy during the ICU stay. A small proportion of patients did not have recorded antithrombotic therapy, which may be explained by contraindications or high bleeding risk, delayed initiation after intravenous thrombolysis or endovascular treatment, severe clinical instability, treatment limitations, or incomplete medication documentation in the retrospective database. Higher RAR values were associated with worse clinical outcomes, including higher hospital and ICU mortality rates, longer ICU stays, and higher 30-day and 90-day mortality rates.

**Table 1:** Baseline characteristics of AIS patients according to RAR tertiles.

Variables	Total (*n* = 1,266)	T1 (*n* = 422)	T2 (*n* = 421)	T3 (*n* = 423)	*p*
Age (year)	72.00 (61.00, 82.00)	70.00 (60.00, 80.00)	75.00 (64.00, 84.00)	71.00 (58.00, 81.00)	< 0.001
Gender, *n* (%)					0.029
Female	643 (50.79)	210 (49.76)	197 (46.79)	236 (55.79)	
Male	623 (49.21)	212 (50.24)	224 (53.21)	187 (44.21)	
Race, *n* (%)					0.027
White	805 (63.59)	263 (62.32)	272 (64.61)	270 (63.83)	
Black	127 (10.03)	41 (9.72)	30 (7.13)	56 (13.24)	
Other	334 (26.38)	118 (27.96)	119 (28.27)	97 (22.93)	
Vital signs					
Heart rate (beats/min)	82.50 (72.00, 98.00)	79.00 (68.00, 89.00)	80.00 (71.00, 96.00)	89.00 (77.00, 105.00)	< 0.001
MBP (mmHg)	90.00 (77.00, 103.00)	94.00 (82.25, 109.00)	91.00 (78.00, 103.00)	85.00 (73.00, 99.00)	< 0.001
Resp rate (times/min)	18.00 (15.00, 22.00)	17.00 (15.00, 20.00)	18.00 (15.00, 22.00)	19.00 (16.00, 24.00)	< 0.001
GCS	14.00 (11.00, 15.00)	14.00 (11.00, 15.00)	14.00 (11.00, 15.00)	14.00 (11.00, 15.00)	0.126
Comorbidity					
Hypertension, n (%)	956 (75.51)	329 (77.96)	316 (75.06)	311 (73.52)	0.313
Diabetes, *n* (%)	403 (31.83)	118 (27.96)	126 (29.93)	159 (37.59)	0.006
AF *n* (%)	558 (44.08)	168 (39.81)	204 (48.46)	186 (43.97)	0.041
Myocardial infarct, *n* (%)	262 (20.70)	74 (17.54)	87 (20.67)	101 (23.88)	0.075
Chronic pulmonary disease, *n* (%)	269 (21.25)	70 (16.59)	92 (21.85)	107 (25.3)	0.008
Renal disease, *n* (%)	257 (20.30)	49 (11.61)	95 (22.57)	113 (26.71)	< 0.001
Laboratory tests					
WBC (K/μl)	10.15 (7.80, 13.80)	9.55 (7.60, 12.40)	10.40 (7.90, 13.80)	10.80 (7.75, 15.30)	< 0.001
RBC (K/μL)	3.85 (3.27, 4.39)	4.17 (3.72, 4.62)	3.86 (3.47, 4.37)	3.35 (2.94, 3.97)	< 0.001
Hemoglobin (g/dL)	11.50 (9.53, 13.10)	12.70 (11.40, 13.90)	11.60 (10.30, 13.10)	9.70 (8.30, 11.30)	< 0.001
Platelet (K/μL)	203.00 (153.00, 268.00)	204.00 (165.25, 257.00)	205.00 (163.00, 278.00)	198.00 (127.00, 281.50)	0.066
RDW (%)	14.10 (13.20, 15.40)	13.20 (12.70, 13.70)	14.10 (13.40, 14.90)	16.00 (14.60, 18.10)	< 0.001
Creatinine (mg/dL)	0.90 (0.70, 1.30)	0.90 (0.70, 1.10)	1.00 (0.80, 1.30)	1.10 (0.70, 1.60)	< 0.001
BUN (mg/dL)	19.00 (13.00, 27.00)	16.00 (12.00, 22.00)	18.00 (14.00, 26.00)	24.00 (15.00, 40.00)	< 0.001
ALT (IU/L)	20.00 (13.00, 33.00)	18.00 (13.00, 26.75)	19.00 (13.00, 36.00)	23.00 (14.00, 45.50)	< 0.001
AST (IU/L)	27.00 (19.00, 48.00)	23.00 (18.00, 33.00)	26.00 (19.00, 46.00)	38.00 (23.00, 69.50)	< 0.001
Albumin (g/dL)	3.50 (3.00, 3.90)	4.00 (3.80, 4.20)	3.50 (3.30, 3.70)	2.80 (2.50, 3.10)	< 0.001
Glucose (mg/dL)	124.00 (102.00, 158.00)	118.00 (100.25, 148.00)	124.00 (102.00, 153.00)	130.00 (105.00, 169.50)	0.011
Potassium (mEq/L)	4.10 (3.70, 4.50)	4.10 (3.70, 4.40)	4.10 (3.80, 4.50)	4.10 (3.70, 4.60)	0.449
Sodium (mEq/L)	139.00 (136.00, 141.00)	140.00 (137.00, 141.75)	139.00 (136.00, 141.00)	139.00 (136.00, 142.00)	0.126
PT (s)	13.20 (12.00, 15.50)	12.50 (11.60, 14.00)	13.10 (11.90, 15.20)	14.50 (12.75, 17.20)	< 0.001
PTT (s)	29.50 (26.20, 34.90)	29.50 (26.30, 34.38)	28.80 (25.80, 34.00)	30.10 (26.60, 35.85)	0.074
INR	1.20 (1.10, 1.40)	1.10 (1.10, 1.30)	1.20 (1.10, 1.40)	1.30 (1.20, 1.60)	< 0.001
Treatment					
Mechanical thrombectomy, *n* (%)	93 (7.35)	27 (6.4)	41 (9.74)	25 (5.91)	0.068
rtPA, n (%)	84 (6.64)	21 (4.98)	18 (4.28)	45 (10.64)	< 0.001
Antiplatelet, *n* (%)	175 (13.82)	67 (15.88)	61 (14.49)	47 (11.11)	0.119
Anticoagulation, *n* (%)	922 (72.83)	292 (69.19)	309 (73.4)	321 (75.89)	0.087
Outcomes					
Pneumonia	175 (13.82)	30 (7.11)	53 (12.59)	92 (21.75)	< 0.001
Hospital stay (day)	10.57 (6.12, 17.34)	8.15 (4.92, 14.34)	10.11 (5.99, 16.63)	13.81 (8.29, 23.22)	< 0.001
Hospital mortality, *n* (%)	211 (16.67)	38 (9)	69 (16.39)	104 (24.59)	< 0.001
ICU stay (day)	3.99 (2.12, 7.75)	3.24 (1.88, 5.76)	3.83 (2.08, 7.45)	5.23 (2.52, 9.03)	< 0.001
ICU mortality, *n* (%)	135 (10.66)	27 (6.4)	41 (9.74)	67 (15.84)	< 0.001
30-day mortality, *n* (%)	288 (22.75)	51 (12.09)	101 (23.99)	136 (32.15)	< 0.001
90-day mortality, *n* (%)	378 (29.86)	67 (15.88)	139 (33.02)	172 (40.66)	< 0.001

MBP, mean blood pressure; GCS, Glasgow Coma Scale; AF, atrial fibrillation; WBC, white blood cell count; RBC, red blood cell count; RDW, red blood cell distribution width; BUN, blood urea nitrogen; ALT, alanine aminotransferase; AST, aspartate aminotransferase; PT, prothrombin time; PTT, partial thromboplastin time; INR, international normalized ratio; rtPA, recombinant tissue plasminogen activator; ICU, intensive care unit.

**Figure 2:**
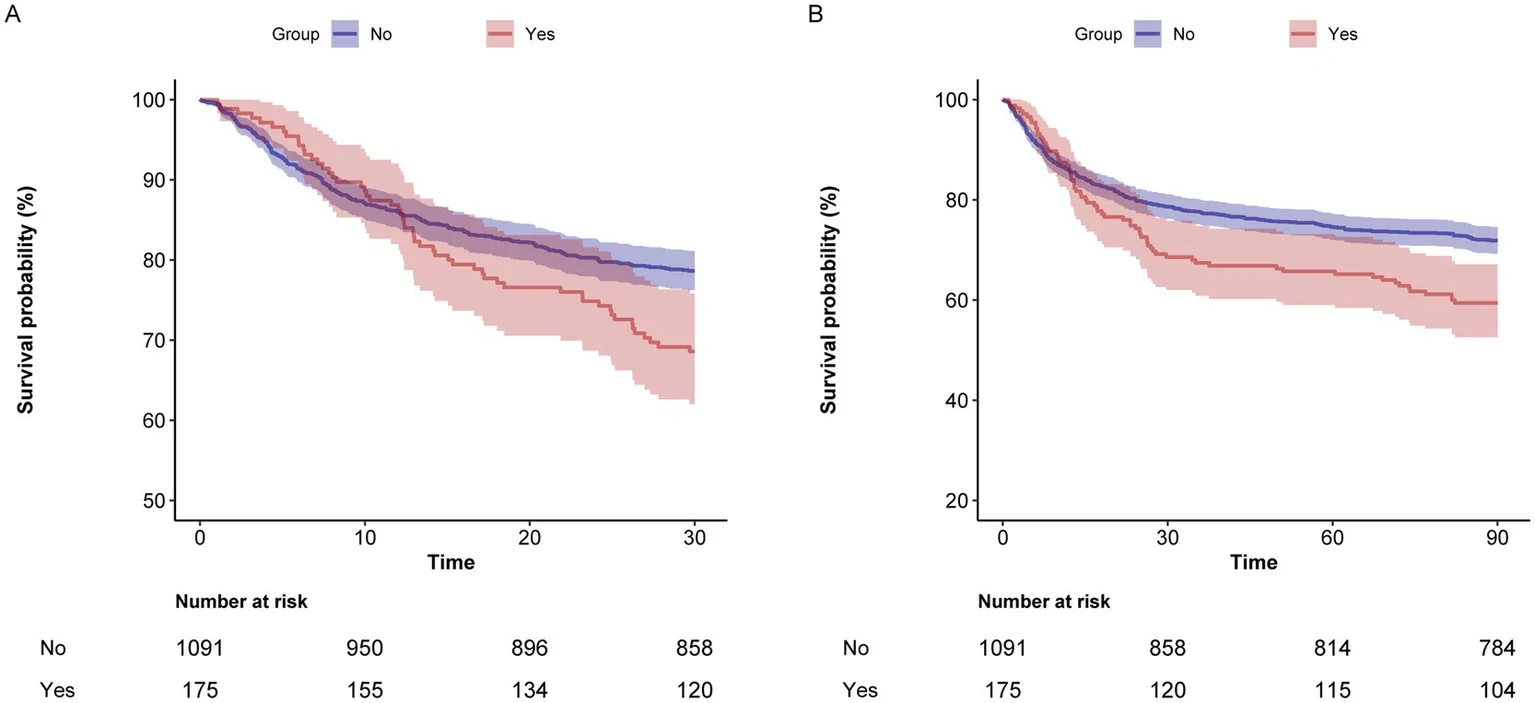
Kaplan–Meier survival curves for 30-day and 90-day mortality in MIMIC IV Cohort. **(A)** 30-day mortality; **(B)** 90-day mortality.

Among the 1,266 AIS patients, 175 developed pneumonia (Table 2). The pneumonia group was significantly younger (*p* = 0.04) and had higher heart rates, respiratory rates, and lower MBP compared to the non-pneumonia group (*p* < 0.001). Additionally, the pneumonia group had higher levels of white blood cells, creatinine, BUN, ALT, and AST (p < 0.001). RAR was significantly higher in the pneumonia group (p < 0.001). A larger proportion of pneumonia patients received rtPA (10.29%) compared to those without pneumonia (6.05%) (p = 0.04), while the use of mechanical thrombectomy and anticoagulation therapy was similar between the groups. Figure 2 shows that patients with pneumonia had significantly lower 30-day and 90-day survival probabilities compared to those without pneumonia.

**Table 2:** Baseline characteristics of the pneumonia and non-pneumonia groups in MIMIC-IV database.

Variables	Total (*n* = 1,266)	Non- pneumonia (*n* = 1,091)	Pneumonia (*n* = 175)	*p*
Age (year)	72.00 (61.00, 82.00)	73.00 (61.00, 82.00)	69.00 (58.00, 82.00)	0.04
Gender, *n* (%)				0.15
Female	643 (50.79)	563 (51.6)	80 (45.71)	
Male	623 (49.21)	528 (48.4)	95 (54.29)	
Race, *n* (%)				0.56
White	805 (63.59)	700 (64.16)	105 (60)	
Black	127 (10.03)	107 (9.81)	20 (11.43)	
Other	334 (26.38)	284 (26.03)	50 (28.57)	
Vital signs				
Heart rate (beats/min)	82.50 (72.00, 98.00)	81.00 (71.00, 96.00)	92.00 (77.50, 106.50)	< 0.001
MBP (mmHg)	90.00 (77.00, 103.00)	91.00 (78.00, 103.00)	87.00 (75.00, 101.00)	0.03
Resp rate (times/min)	18.00 (15.00, 22.00)	18.00 (15.00, 22.00)	20.00 (16.00, 26.00)	< 0.001
GCS	14.00 (11.00, 15.00)	14.00 (11.00, 15.00)	15.00 (11.00, 15.00)	0.06
Comorbidity				
Hypertension, *n* (%)	956 (75.51)	824 (75.53)	132 (75.43)	0.98
Diabetes, *n* (%)	403 (31.83)	353 (32.36)	50 (28.57)	0.32
AF *n* (%)	558 (44.08)	479 (43.9)	79 (45.14)	0.76
Myocardial infarct, *n* (%)	262 (20.70)	225 (20.62)	37 (21.14)	0.88
Chronic pulmonary disease, *n* (%)	269 (21.25)	222 (20.35)	47 (26.86)	0.05
Renal disease, *n* (%)	257 (20.30)	212 (19.43)	45 (25.71)	0.06
Laboratory tests				
WBC (K/μL)	10.15 (7.80, 13.80)	9.90 (7.70, 13.45)	12.10 (8.70, 16.70)	< 0.001
RBC (K/μL)	3.85 (3.27, 4.39)	3.86 (3.29, 4.40)	3.77 (3.14, 4.32)	0.11
Hemoglobin (g/dL)	11.50 (9.53, 13.10)	11.50 (9.70, 13.20)	11.20 (9.00, 12.90)	0.06
Platelet (K/μL)	203.00 (153.00, 268.00)	204.00 (156.00, 267.50)	192.00 (131.00, 273.50)	0.09
Creatinine (mg/Dl)	0.90 (0.70, 1.30)	0.90 (0.70, 1.30)	1.00 (0.80, 1.60)	0.001
BUN (mg/dL)	19.00 (13.00, 27.00)	18.00 (13.00, 26.00)	21.00 (15.00, 36.00)	< 0.001
ALT (IU/L)	20.00 (13.00, 33.00)	19.00 (13.00, 32.00)	25.00 (15.50, 46.00)	< 0.001
AST (IU/L)	27.00 (19.00, 48.00)	26.00 (19.00, 45.50)	39.00 (23.00, 67.00)	< 0.001
RAR	4.07 (3.54, 5.11)	4.00 (3.50, 4.97)	4.73 (3.94, 5.92)	< 0.001
Glucose (mg/dL)	124.00 (102.00, 158.00)	122.00 (101.00, 153.00)	136.00 (109.50, 177.50)	< 0.001
Potassium (mEq/L)	4.10 (3.70, 4.50)	4.10 (3.80, 4.50)	4.00 (3.70, 4.45)	0.35
Sodium (mEq/L)	139.00 (136.00, 141.00)	139.00 (136.00, 141.50)	139.00 (136.50, 141.00)	0.85
PT (s)	13.20 (12.00, 15.50)	13.10 (11.90, 15.20)	14.50 (12.55, 17.35)	< 0.001
PTT (s)	29.50 (26.20, 34.90)	29.50 (26.20, 34.80)	29.60 (26.60, 35.10)	0.55
INR	1.20 (1.10, 1.40)	1.20 (1.10, 1.40)	1.30 (1.10, 1.60)	< 0.001
Treatment				
Mechanical thrombectomy, *n* (%)	93 (7.35)	83 (7.61)	10 (5.71)	0.37
rtPA, *n* (%)	84 (6.64)	66 (6.05)	18 (10.29)	0.04
Antiplatelet, *n* (%)	175 (13.82)	150 (13.75)	25 (14.29)	0.85
Anticoagulation, *n* (%)	922 (72.83)	790 (72.41)	132 (75.43)	0.41

MBP, mean blood pressure; GCS, Glasgow Coma Scale; AF, atrial fibrillation; WBC, white blood cell count; RBC, red blood cell count; RDW, red blood cell distribution width; BUN, blood urea nitrogen; ALT, alanine aminotransferase; AST, aspartate aminotransferase; PT, prothrombin time; PTT, partial thromboplastin time; INR, international normalized ratio; rtPA, recombinant tissue plasminogen activator.

### 3.2 Association between RAR and pneumonia

Covariate selection for the models was based on variables that showed significant differences in Table 2. Candidate covariates were assessed for multicollinearity using VIF analysis (Supplementary Table S2). Severe multicollinearity was identified between PT and INR (VIF > 55); therefore, they were not simultaneously considered in the final model. The univariate regression results for these variables are presented in Supplementary Table S3. Table 3 presents the association between RAR and pneumonia in both the MIMIC-IV and XMHH cohorts. In the MIMIC-IV cohort, both continuous RAR and RAR tertiles were significantly associated with pneumonia risk. For continuous RAR, each unit increase was associated with a 24% higher risk of pneumonia (OR: 1.24, 95% CI: 1.14–1.35, *p* < 0.001), and the association remained significant after adjusting for age, gender, vital signs, and laboratory results (OR: 1.11, 95% CI: 1.02–1.21, *p* = 0.019). In tertiles, patients in the T3 group had the highest risk (OR: 2.11, 95% CI: 1.30–3.41, *p* = 0.003). Sensitivity analyses were generally consistent with the primary findings. Rubin-pooled estimates across five imputed datasets remained significant for continuous RAR and for T3 versus T1 in the fully adjusted model, whereas the complete-case analysis showed a similar direction of association but attenuated categorical estimates (Supplementary Tables S4, S5). Additional adjustment for mechanical thrombectomy also did not materially alter the associations in either cohort (Supplementary Table S6).

**Table 3:** Analysis for association between RAR and pneumonia.

Variables	Model 1		Model 2		Model 3	
	OR (95%CI)	*p*	OR (95%CI)	*p*	OR (95%CI)	*p*
MIMIC IV 3.1 cohort						
RAR (Continuous)	1.24 (1.14, 1.35)	<0.001	1.14 (1.05, 1.24)	0.002	1.11 (1.02, 1.21)	0.019
RAR (Tertiles)						
T1	1(Ref)		1(Ref)		1(Ref)	
T2	1.88 (1.18, 3.01)	0.008	1.67 (1.04, 2.71)	0.035	1.56 (0.96, 2.53)	0.072
T3	3.63 (2.35, 5.62)	<0.001	2.57 (1.62, 4.09)	<0.001	2.11 (1.3, 3.41)	0.003
*P* for trend		<0.001		<0.001		0.002
XMHH cohort						
RAR (Continuous)	3.5 (1.83, 6.69)	<0.001	3.6 (1.84, 7.04)	<0.001	2.65 (1.25, 5.64)	0.011
RAR (Tertiles)						
T1	1(Ref)		1(Ref)		1(Ref)	
T2	1.84 (0.59, 5.71)	0.291	1.49 (0.44, 5)	0.518	1.39 (0.39, 4.95)	0.608
T3	6.39 (2.34, 17.47)	<0.001	5.57 (1.86, 16.7)	0.002	3.94 (1.27, 12.27)	0.018
*P* for trend		<0.001		0.001		0.013

Model 1: No adjustment for potential confounders.

Model 2: Adjusted for age, gender, heart rate, mean blood pressure (MBP), and respiratory rate.

Model 3: Adjusted for age, gender, heart rate, MBP, respiratory rate, white blood cell count (WBC), Glasgow Coma Scale (GCS), creatinine, blood urea nitrogen (BUN), alanine aminotransferase (ALT), aspartate aminotransferase (AST), glucose, and use of rtPA.

In the XMHH cohort, higher continuous RAR was also associated with an increased risk of pneumonia (OR: 3.50, 95% CI: 1.83–6.69, p < 0.001). After adjustment, the OR remained significant at 2.65 (95% CI: 1.25–5.64, *p* = 0.011). For RAR tertiles, the highest risk was in the T3 group (OR: 6.39, 95% CI: 2.34–17.47, *p* < 0.001), and the association remained robust after adjustment (OR: 3.94, 95% CI: 1.27–12.27, *p* = 0.018).

### 3.3 Non-linear relationship between RAR and pneumonia

**Figure 3:**
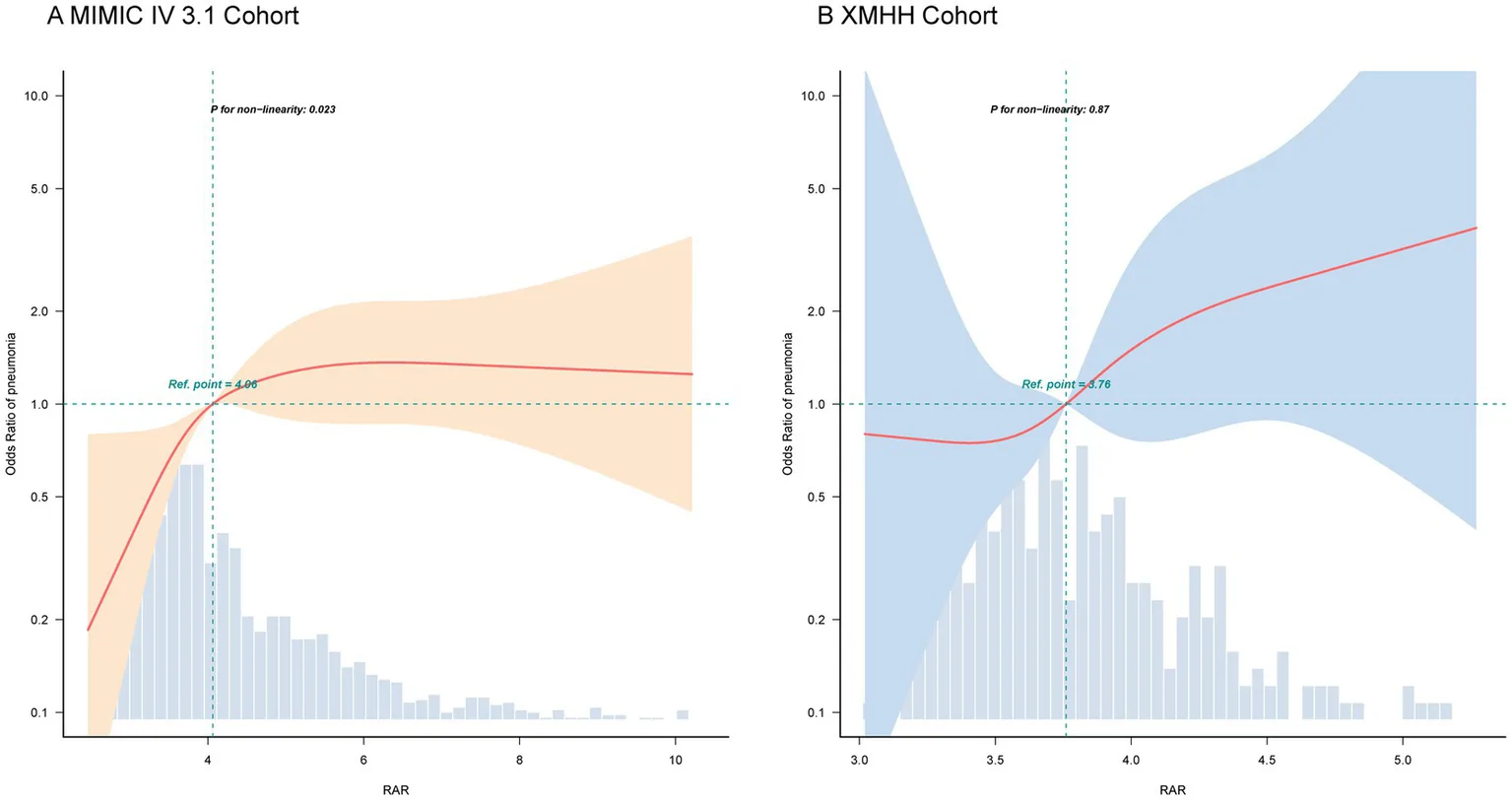
Restricted cubic spline analysis of the association between RAR and pneumonia. **(A)** MIMIC-IV cohort; **(B)** XMHH cohort. The red line represents the adjusted odds ratio, and the shaded area represents the 95% confidence interval. The horizontal dashed line indicates an odds ratio of 1.0, and the vertical dashed line indicates the reference RAR value. The distribution of RAR is shown by the histogram. Although the association was nonlinear in the MIMIC-IV cohort, no distinct and reproducible clinical risk threshold was identified across the two cohorts.

Figure 3 presents the RCS analysis for RAR and pneumonia in both the MIMIC-IV and XMHH cohorts. In the MIMIC-IV cohort, the OR for pneumonia increased with higher RAR values, but the association showed non-linearity (P for non-linearity = 0.023), indicating that the effect of RAR on pneumonia risk changes at higher RAR levels. In the XMHH cohort, the non-linear relationship was less pronounced (P for non-linearity = 0.87), suggesting a more stable and linear association across the range of RAR values.

### 3.4 Discriminatory performance of RAR

**Figure 4:**
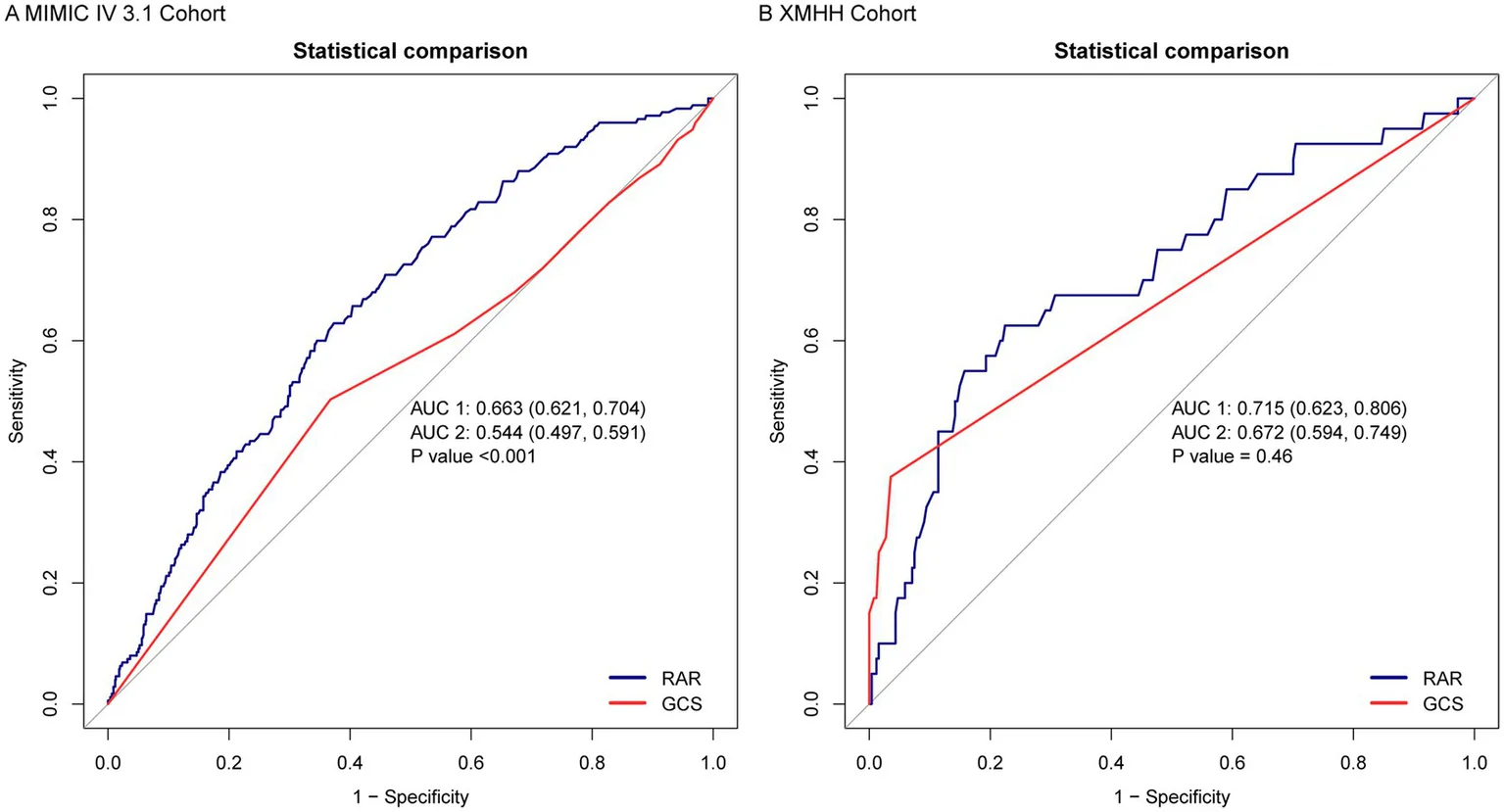
ROC curve comparison between RAR and GCS. **(A)** MIMIC-IV cohort; **(B)** XMHH cohort.

Figure 4 compares the discriminatory performance of RAR and GCS for pneumonia in both cohorts. In the MIMIC-IV cohort, RAR achieved an AUC of 0.663 (95% CI: 0.621–0.704), which was significantly higher than that of GCS (AUC 0.544, 95% CI: 0.497–0.591; p < 0.001). In the XMHH cohort, RAR also showed a higher AUC than GCS [0.715 (95% CI: 0.623–0.806) vs. 0.672 (95% CI: 0.594–0.749)], although the difference was not statistically significant (*p* = 0.46). Additional analyses showed that RAR outperformed RDW alone in the MIMIC-IV cohort, whereas albumin had a slightly higher AUC. In the XMHH cohort, RAR had the highest AUC among RAR, RDW, and albumin, but the pairwise differences were not statistically significant (Supplementary Figure S2 and Supplementary Table S7). Overall, RAR demonstrated modest discriminatory ability, with evidence of better performance than GCS and RDW in the MIMIC-IV cohort, but without consistently superior performance across both cohorts.

### 3.5 Subgroup analysis

**Figure 5:**
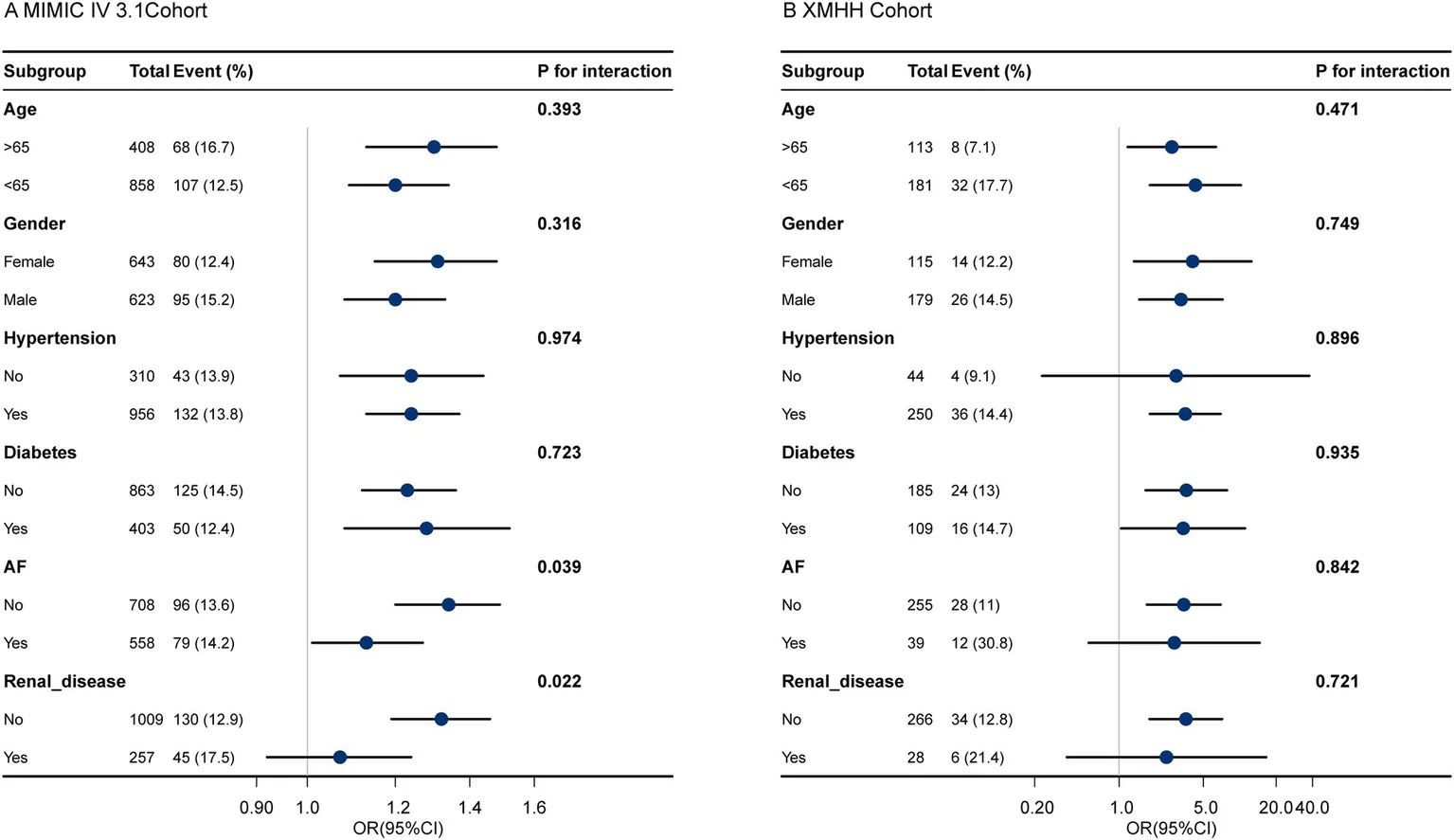
Forest plot of subgroup analysis. **(A)** MIMIC-IV cohort; **(B)** XMHH cohort.

Figure 5 presents the results of the subgroup analysis for the association between RAR and pneumonia in both the MIMIC-IV (Panel A) and XMHH cohorts. In the MIMIC-IV cohort, the association between RAR and pneumonia was consistent across most subgroups, with no significant interactions observed for age (*p* = 0.393), gender (*p* = 0.316), hypertension (*p* = 0.974), or diabetes (*p* = 0.723). However, the association was stronger in patients without AF (P for interaction = 0.039) or renal disease (P for interaction = 0.022), suggesting that these factors may influence the effect of RAR on pneumonia risk. In the XMHH cohort, the association between RAR and pneumonia was not significantly modified by age (*p* = 0.471), gender (*p* = 0.749), hypertension (*p* = 0.896), or diabetes (*p* = 0.935). Similarly, no significant interactions were found for AF (*p* = 0.842) or renal disease (*p* = 0.721). The lack of significant subgroup interactions in this cohort suggests that the association between RAR and pneumonia is more consistent across different demographic and clinical characteristics in the XMHH cohort.

### 3.6 *E*-value analysis

*E*-value analysis was conducted to assess the potential impact of unmeasured confounders. Supplementary Figure S3 shows that the *E*-value for the MIMIC-IV cohort was 1.46, indicating some instability in the association between RAR and pneumonia. In contrast, the *E*-value for the XMHH cohort was 4.74, suggesting a more stable and robust association. These results indicate that while the MIMIC-IV cohort’s association may be influenced by unmeasured confounders, the XMHH cohort’s findings are more robust.

### 3.7 Exploratory nomogram development

In the multivariable analysis including RAR and all Model 3 covariates, RAR, heart rate, WBC, BUN, and ALT remained significantly associated with pneumonia, while respiratory rate showed borderline significance (Supplementary Table S8). Based on these findings and clinical relevance, an exploratory nomogram incorporating age, RAR, heart rate, respiratory rate, WBC, BUN, and ALT was developed. The nomogram achieved an AUC of 0.696 (95% CI 0.652–0.741) in the MIMIC-IV cohort. The bootstrap-corrected calibration curve showed reasonable agreement between predicted and observed probabilities, and decision curve analysis suggested potential clinical net benefit across a range of threshold probabilities (Supplementary Figure S4).

## 4 Discussion

This study investigated the association between RAR and pneumonia during ICU stay among patients with AIS using two independent cohorts. Higher RAR was consistently associated with a greater likelihood of pneumonia in both the MIMIC-IV and XMHH cohorts. However, given the retrospective design, the modest discriminatory performance, and the uncertainty regarding the exact timing of pneumonia onset in the MIMIC-IV cohort, these findings should be interpreted as evidence of association rather than definitive prediction or causality.

The pneumonia outcome was not uniformly adjudicated across the two cohorts. In MIMIC-IV, pneumonia was identified using diagnostic codes recorded during hospitalization, and the timing of onset relative to admission and RAR measurement could not be reliably determined. Therefore, some cases may have represented pneumonia already present at admission rather than newly developed during the ICU stay. In contrast, pneumonia in the XMHH cohort was based on clinical diagnosis and medical record review, with pre-existing pneumonia excluded. These differences in outcome ascertainment may have introduced misclassification and may partly explain the variation in effect estimates and discriminatory performance between cohorts. Although the direction of the association was consistent, the findings should be interpreted as an association between RAR and pneumonia documented during hospitalization rather than as evidence that RAR predicts newly developed stroke-associated pneumonia. Future prospective studies should use standardized diagnostic criteria and clearly adjudicated pneumonia onset.

The association between RDW, albumin, and stroke outcomes has been well-documented in existing literature, with RDW reflecting systemic inflammation, oxidative stress, and endothelial dysfunction—key factors in both stroke pathophysiology and pneumonia development (8, 28, 29). RDW represents the degree of variability in red blood cell size, and an elevated RDW reflects dysregulated erythropoiesis, which is closely linked to increased systemic inflammation. In stroke patients, this heightened inflammation, due to both the ischemic insult and the subsequent neuroinflammatory response, may contribute to lung injury and increased vulnerability to pneumonia. Inflammatory mediators, such as cytokines, may compromise lung function by increasing vascular permeability and promoting fluid accumulation in the lungs, creating a favorable environment for infection (30). Furthermore, endothelial dysfunction, a hallmark of stroke-related injury, can disrupt the blood–brain barrier and other vascular barriers, including those in the lungs, allowing pathogens to translocate more easily into the pulmonary system, increasing pneumonia risk (31).

Albumin, a known marker of nutritional status and systemic inflammation, also plays a critical role in immune function (11). Low albumin levels are associated with a suppressed immune response, as albumin has anti-inflammatory properties that help regulate immune cell activity and maintain endothelial integrity (32). In AIS patients, albumin levels are often reduced due to poor nutritional intake, hypercatabolism, and liver dysfunction following the stroke. The reduction in albumin not only increases susceptibility to infection but also impairs the body’s ability to mount an effective immune response. Albumin’s protective role in maintaining vascular integrity also plays a key part in preventing infections by ensuring that the endothelial barrier remains intact (33). Thus, low albumin levels, which are commonly observed in AIS patients, may facilitate bacterial infiltration and contribute to pneumonia development.

By combining RDW and albumin into the RAR, this biomarker captures both the inflammatory burden (through RDW) and the nutritional and immune deficiencies (through albumin). Elevated RAR thus serves as a comprehensive reflection of a patient’s systemic inflammatory status and immune function, two factors that are critical in the development of pneumonia. An increased RAR may signal that the patient is in a heightened state of inflammation and has a compromised immune system, both of which predispose them to infections such as pneumonia. In this context, RAR serves as a surrogate for the combined effects of inflammation and immune suppression, indicating a patient at higher risk of pneumonia.

The present study focused on ICU patients because they represent a high-risk subgroup with greater neurological and systemic disease severity and a higher likelihood of pneumonia-related complications. However, this restriction also limits the generalizability of the findings, and the observed association may not apply to patients with milder AIS managed outside the ICU. The clinical utility of RAR should be interpreted cautiously because its discriminatory performance was modest, with AUC values of 0.663 in the MIMIC-IV cohort and 0.715 in the XMHH cohort. These values do not support its use as a standalone predictive marker. Moreover, comparison with GCS alone is insufficient to establish the incremental value of RAR, because validated stroke-associated pneumonia prediction models, including A^2^DS^2^, AIS-APS, and ISAN, already incorporate multiple established clinical risk factors. Several variables required to calculate these scores, such as NIHSS, dysphagia, and prestroke functional status, were not consistently available across the two cohorts. Therefore, we could not directly compare RAR with these established models or determine whether it provides additional predictive information beyond conventional clinical assessment. Future prospective studies should evaluate whether adding RAR to validated SAP models improves discrimination, calibration, reclassification, and clinical decision-making.

Patients with higher RAR also had longer ICU and hospital stays, which may be clinically relevant to pneumonia development. Longer hospitalization may increase exposure to invasive procedures, prolonged immobilization, aspiration risk, mechanical ventilation, and hospital-acquired pathogens, thereby increasing the likelihood of pneumonia. Conversely, pneumonia itself may prolong ICU and hospital stays by increasing clinical complexity, delaying recovery, and requiring additional respiratory support or antimicrobial therapy. Therefore, the association between length of stay and pneumonia is likely bidirectional. Because ICU and hospital length of stay may occur after pneumonia onset and may partly reflect downstream consequences of pneumonia, these variables were not included as adjustment covariates in the main regression models to avoid potential overadjustment or post-outcome adjustment bias. Future prospective studies with precise time-to-event data are needed to better clarify the temporal relationship among RAR, pneumonia, and length of stay.

In addition to the overall findings, we observed significant interactions between RAR and pneumonia risk in specific subgroups. Interestingly, the relationship between RAR and pneumonia risk was more pronounced in patients without AF or renal disease. In the AF subgroup, the effect of RAR on pneumonia risk was less significant, which may be due to the chronic inflammatory state and hemodynamic instability often seen in these patients, which could potentially obscure the predictive value of RAR (34). Similarly, in patients with renal disease, the association between RAR and pneumonia was weaker, likely due to the immune dysfunction and chronic inflammation commonly observed in these patients, which may reduce the effectiveness of RAR as a predictor (35). These findings suggest that RAR could be a particularly useful predictor of pneumonia risk in patients without AF or renal disease, enabling more targeted preventive strategies in these high-risk individuals. The subgroup analyses were exploratory. Although nominal interactions were observed for atrial fibrillation and renal disease in the MIMIC-IV cohort, these findings were not replicated in the XMHH cohort, and no adjustment for multiple testing was performed. Therefore, these results may reflect chance findings and should not be considered definitive evidence of effect modification.

The relatively low *E*-value of 1.46 in the MIMIC-IV cohort indicates that the observed association was sensitive to unmeasured confounding. A moderately strong unmeasured factor associated with both RAR and pneumonia could potentially explain away the observed effect estimate. This concern is particularly relevant because several major predictors of stroke-associated pneumonia, including NIHSS, dysphagia, aspiration risk, feeding tube placement, and mechanical ventilation, were not consistently available. Although the higher *E*-value in the XMHH cohort suggests greater robustness of its point estimate, the smaller sample size and wider confidence intervals should also be considered. Therefore, the *E*-value findings do not eliminate concerns regarding residual confounding, and the overall association should be interpreted cautiously.

Although this study provides valuable insights, several limitations should be noted. First, the retrospective design of both the MIMIC-IV and XMHH cohorts inherently carries the risk of information bias, as the data were originally collected for clinical purposes and may not have captured all relevant variables. Although RAR was calculated using RDW and albumin values obtained within the first 24 h of ICU admission, the exact timing of pneumonia onset relative to blood sampling could not be reliably determined for all patients in the MIMIC-IV cohort. Therefore, reverse causation cannot be excluded, and elevated RAR may partly reflect existing infection, systemic inflammation, or overall illness severity rather than strictly predicting subsequent pneumonia. Accordingly, our findings should be interpreted as an association between early RAR and pneumonia during ICU stay rather than definitive evidence of prediction or causality. Second, selection bias may have arisen from the exclusion of a substantial proportion of potentially eligible patients because of missing RDW or albumin measurements. In the MIMIC-IV cohort, nearly half of the initially identified AIS population was excluded, predominantly because albumin was not measured. Because laboratory testing may be more likely in patients with greater disease severity, suspected infection, nutritional impairment, or more intensive clinical monitoring, the included cohort may differ systematically from patients without these measurements. Therefore, the findings may not be fully generalizable to all ICU patients with AIS. Third, residual confounding remains a concern. Stroke severity is one of the strongest determinants of stroke-associated pneumonia; however, NIHSS scores were not consistently available in MIMIC-IV and could not be included in the analyses or harmonized across the two cohorts. Although GCS was adjusted for as an indicator of consciousness level, it does not comprehensively capture focal neurological deficits and cannot substitute for NIHSS. In addition, pre-existing respiratory infections were not uniformly identifiable across the two cohorts, and patients with severe hematological or hepatic diseases were not specifically excluded. These conditions may have influenced RDW, albumin levels, and pneumonia risk, thereby affecting the observed association. Other unmeasured factors related to ICU care and treatment practices may also have contributed to residual confounding, as reflected by the relatively low *E*-value in the MIMIC-IV cohort. Finally, this study assessed RAR based on RDW and albumin values obtained within the first 24 h of ICU admission and did not evaluate dynamic changes in RAR during the subsequent disease course. Therefore, whether longitudinal changes in RAR provide additional predictive value for pneumonia risk remains unclear. Future prospective studies with standardized repeated measurements and detailed neuroimaging information are needed to further clarify the temporal trajectory, clinical utility, and location-specific relevance of RAR.

In conclusion, higher RAR was independently associated with pneumonia during ICU stay among patients with AIS in two independent cohorts. Given its low cost and routine availability, RAR may serve as an adjunctive marker for early pneumonia risk stratification. However, its discriminatory performance was modest, and its incremental clinical utility beyond established stroke-associated pneumonia predictors remains uncertain. Prospective studies with precise pneumonia onset adjudication, standardized serial biomarker measurements, and comprehensive clinical predictors are needed to validate its clinical relevance.

## Data Availability

Publicly available de-identified data from the MIMIC-IV database were used in this study and can be accessed through PhysioNet after completion of the required credentialing and data use agreement. The de-identified data from the XMHH cohort are available from the corresponding authors upon reasonable request, subject to approval by the Ethics Committee of Xiamen Humanity Hospital and applicable institutional and privacy regulations.
