# Supplementary material for: Association between red blood cell distribution width to albumin ratio and pneumonia risk in acute ischemic stroke: evidence from two independent cohorts
Source: Front Nutr. 2026 Aug 17;13:1847555. doi: 10.3389/fnut.2026.1847555 (PMC13526672; doi:10.3389/fnut.2026.1847555)
Supplement: Supplementary file 1 [file Table_1.DOCX]

Table S1. Comparison of baseline characteristics between the MIMIC-IV and XMHH cohorts

| **Variables** | **MIMIC-IV cohort (n=1266)** | **XMHH cohort (n=294)** | **P** |
| --- | --- | --- | --- |
| Age (year) | 72 (61, 82) | 69 (59, 76) | <0.001 |
| Heart rate (beats/min) | 82.50 (72.00, 98.00) | 76.00 (67.00, 84.75) | <0.001 |
| MBP (mmHg) | 90.00 (77.12, 103.00) | 110.00 (101.00, 121.00) | <0.001 |
| Respiratory rate (times/min) | 18.00 (15.00, 22.00) | 19.00 (18.00, 20.00) | 0.033 |
| GCS | 14.00 (11.00, 15.00) | 15.00 (15.00, 15.00) | <0.001 |
| WBC (K/μL) | 10.15 (7.80, 13.80) | 7.33 (6.16, 9.00) | <0.001 |
| RBC (10⁶/μL) | 3.85 (3.27, 4.39) | 4.62 (4.23, 5.00) | <0.001 |
| Hemoglobin (g/dL) | 11.50 (9.53, 13.10) | 13.90 (12.53, 15.17) | <0.001 |
| Platelet (K/μL) | 203.00 (153.00, 268.00) | 222.00 (186.00, 269.00) | <0.001 |
| RDW (%) | 14.10 (13.20, 15.40) | 15.25 (14.54, 15.90) | <0.001 |
| Creatinine (mg/dL) | 0.90 (0.70, 1.30) | 0.84 (0.70, 1.00) | <0.001 |
| BUN (mg/dL) | 19.00 (13.00, 27.00) | 15.80 (12.86, 20.68) | <0.001 |
| ALT (IU/L) | 20.00 (13.00, 33.00) | 16.45 (11.80, 25.48) | <0.001 |
| AST (IU/L) | 27.00 (19.00, 48.00) | 21.70 (17.95, 27.48) | <0.001 |
| Albumin (g/dL) | 3.50 (3.00, 3.90) | 4.05 (3.79, 4.24) | <0.001 |
| RAR | 4.07 (3.54, 5.11) | 3.77 (3.52, 4.08) | <0.001 |
| Glucose (mg/dL) | 124.00 (102.00, 158.00) | 123.96 (101.98, 167.48) | 0.581 |
| Potassium (mEq/L) | 4.10 (3.70, 4.50) | 3.78 (3.54, 3.99) | <0.001 |
| Sodium (mEq/L) | 139.00 (136.00, 141.00) | 138.10 (136.10, 139.70) | <0.001 |
| PT (s) | 13.20 (12.00, 15.50) | 11.80 (11.30, 12.40) | <0.001 |
| PTT (s) | 29.50 (26.20, 34.90) | 27.30 (25.70, 29.40) | <0.001 |
| INR | 1.20 (1.10, 1.40) | 1.03 (0.97, 1.09) | <0.001 |
| Male, n (%) | 623 (49.21) | 179 (60.88) | <0.001 |
| Hypertension, n (%) | 956 (75.51) | 250 (85.03) | <0.001 |
| Diabetes, n (%) | 403 (31.83) | 109 (37.07) | 0.085 |
| Atrial fibrillation, n (%) | 558 (44.08) | 39 (13.27) | <0.001 |
| Myocardial infarction, n (%) | 262 (20.70) | 32 (10.88) | <0.001 |
| Chronic pulmonary disease, n (%) | 269 (21.25) | 2 (0.68) | <0.001 |
| Renal disease, n (%) | 257 (20.30) | 28 (9.52) | <0.001 |
| Mechanical thrombectomy, n (%) | 93 (7.35) | 6 (2.04) | <0.001 |
| rtPA use, n (%) | 84 (6.64) | 49 (16.67) | <0.001 |
| Antiplatelet therapy, n (%) | 175 (13.82) | 257 (87.41) | <0.001 |
| Anticoagulation therapy, n (%) | 922 (72.83) | 71 (24.15) | <0.001 |
| Pneumonia, n (%) | 175 (13.82) | 40 (13.61) | 0.922 |

Abbreviations: MBP, mean blood pressure; GCS, Glasgow Coma Scale; AF, atrial fibrillation; WBC, white blood cell count; RBC, red blood cell count; RDW, red blood cell distribution width; BUN, blood urea nitrogen; ALT, alanine aminotransferase; AST, aspartate aminotransferase; PT, prothrombin time; PTT, partial thromboplastin time; INR, international normalized ratio; rtPA, recombinant tissue plasminogen activator.

Table S2 Variance Inflation Factor (VIF) and collinearity assessment.

| **Variables** | **VIF** | **Colinearity** |
| --- | --- | --- |
| RAR | 1.171 | No |
| Age | 1.127 | No |
| Gender | 1.069 | No |
| Heart rate | 1.267 | No |
| MBP | 1.091 | No |
| Resp rate | 1.208 | No |
| WBC | 1.077 | No |
| GCS | 1.03 | No |
| Creatinine | 1.838 | No |
| BUN | 1.992 | No |
| ALT | 2.467 | No |
| AST | 2.513 | No |
| Glucose | 1.068 | No |
| PT | 55.55 | Yes |
| INR | 55.41 | Yes |
| rtPA | 1.072 | No |

Abbreviations: MBP, mean blood pressure; GCS, Glasgow Coma Scale; WBC, white blood cell count; ALT, alanine aminotransferase; AST, aspartate aminotransferase; BUN, blood urea nitrogen; rtPA, recombinant tissue plasminogen activator; INR, international normalized ratio; PT, prothrombin time.

Table S3 Univariate logistic regression analysis.

| **Variable** | **OR (95%CI)** | ***P*** |
| --- | --- | --- |
| Age | 0.99 (0.98, 1.00) | 0.02 |
| Gender (Male vs Female) | 1.27 (0.92, 1.74) | 0.15 |
| Heart rate | 1.03 (1.02, 1.03) | <0.001 |
| MBP | 0.99 (0.98, 1.00) | 0.03 |
| Resp rate | 1.07 (1.04, 1.09) | <0.001 |
| WBC | 1.05 (1.0, 1.07) | <0.001 |
| GCS | 1.01 (0.96, 1.07) | 0.67 |
| Creatinine | 1.08 (1.01, 1.17) | 0.03 |
| BUN | 1.02 (1.01, 1.02) | <0.001 |
| ALT | 1.0010 (1.0005, 1.0016) | <0.001 |
| AST | 1.00063 (1.0002, 1.0011) | <0.001 |
| Glucose | 1.00223 (1.0006, 1.0038) | 0.01 |
| rtPA | 1.78 (1.03, 3.08) | 0.04 |
| RAR | 1.24 (1.14, 1.35) | <0.001 |

Abbreviations: MBP, mean blood pressure; GCS, Glasgow Coma Scale; WBC, white blood cell count; ALT, alanine aminotransferase; AST, aspartate aminotransferase; BUN, blood urea nitrogen; rtPA, recombinant tissue plasminogen activator; INR, international normalized ratio; PT, prothrombin time; RAR, red blood cell distribution width to albumin ratio.

Table S4. Complete-case sensitivity analysis of the association between RAR and pneumonia in the MIMIC-IV cohort

| **Variables** | **Model 1 OR (95% CI)** | **P** | **Model 2 OR (95% CI)** | **P** | **Model 3 OR (95% CI)** | **P** |
| --- | --- | --- | --- | --- | --- | --- |
| RAR (Continuous) | 1.24 (1.14–1.35) | <0.001 | 1.14 (1.05–1.24) | 0.002 | 1.12 (1.01–1.24) | 0.026 |
| RAR (Tertiles) |  |  |  |  |  |  |
| T1 | 1 (Ref) |  | 1 (Ref) |  | 1 (Ref) |  |
| T2 | 1.87 (1.17–2.99) | 0.009 | 1.64 (1.02–2.66) | 0.043 | 1.32 (0.74–2.36) | 0.352 |
| T3 | 3.65 (2.36–5.66) | <0.001 | 2.59 (1.63–4.13) | <0.001 | 1.71 (0.94–3.09) | 0.078 |
| P for trend |  | <0.001 |  | <0.001 |  | 0.074 |

Model 1: No adjustment for potential confounders.

Model 2: Adjusted for age, gender, heart rate, mean blood pressure (MBP), and respiratory rate.

Model 3: Adjusted for age, gender, heart rate, MBP, respiratory rate, white blood cell count (WBC), Glasgow Coma Scale (GCS), creatinine, blood urea nitrogen (BUN), alanine aminotransferase (ALT), aspartate aminotransferase (AST), glucose, and use of rtPA.

Table S5. Rubin-pooled analysis of the association between RAR and pneumonia in the MIMIC-IV cohort

| **Variables** | **Model 1 OR (95% CI)** | **P** | **Model 2 OR (95% CI)** | **P** | **Model 3 OR (95% CI)** | **P** |
| --- | --- | --- | --- | --- | --- | --- |
| RAR (Continuous) | 1.24 (1.14–1.35) | <0.001 | 1.14 (1.05–1.24) | 0.002 | 1.11 (1.02–1.21) | 0.018 |
| RAR (Tertiles) |  |  |  |  |  |  |
| T1 | 1 (Ref) |  | 1 (Ref) |  | 1 (Ref) |  |
| T2 | 1.87 (1.17–2.99) | 0.009 | 1.67 (1.04–2.71) | 0.035 | 1.57 (0.97–2.54) | 0.069 |
| T3 | 3.65 (2.36–5.66) | <0.001 | 2.61 (1.64–4.15) | <0.001 | 2.11 (1.30–3.42) | 0.003 |
| P for trend |  | <0.001 |  | <0.001 |  | 0.003 |

Model 1: No adjustment for potential confounders.

Model 2: Adjusted for age, gender, heart rate, mean blood pressure (MBP), and respiratory rate.

Model 3: Adjusted for age, gender, heart rate, MBP, respiratory rate, white blood cell count (WBC), Glasgow Coma Scale (GCS), creatinine, blood urea nitrogen (BUN), alanine aminotransferase (ALT), aspartate aminotransferase (AST), glucose, and use of rtPA.

Note: Five datasets were generated using multiple imputation by chained equations, and regression estimates were pooled according to Rubin’s rules. P for trend was calculated by entering RAR tertiles as an ordinal variable.

Table S6. Sensitivity analysis of the association between RAR and pneumonia after additional adjustment for mechanical thrombectomy

| **Variables** | **MIMIC-IV OR (95% CI)** | **P** | **XMHH OR (95% CI)** | **P** |
| --- | --- | --- | --- | --- |
| RAR (Continuous) | 1.11 (1.02–1.20) | 0.020 | 2.84 (1.31–6.17) | 0.008 |
| RAR (Tertiles) |  |  |  |  |
| T1 | 1 (Ref) |  | 1 (Ref) |  |
| T2 | 1.55 (0.95–2.51) | 0.078 | 1.61 (0.45–5.77) | 0.462 |
| T3 | 2.11 (1.30–3.43) | 0.002 | 4.24 (1.31–13.79) | 0.016 |
| P for trend |  | 0.002 |  | 0.013 |

Note: The sensitivity model was adjusted for age, gender, heart rate, mean blood pressure, respiratory rate, white blood cell count, Glasgow Coma Scale, creatinine, blood urea nitrogen, alanine aminotransferase, aspartate aminotransferase, glucose, rtPA use, and mechanical thrombectomy.

Table S7. Comparison of the discriminatory performance of RAR, RDW, and albumin for pneumonia

| **Marker** | **AUC (95% CI)** | **DeLong comparison** | **P** |
| --- | --- | --- | --- |
| **MIMIC-IV cohort** |  |  |  |
| RAR | 0.663 (0.621–0.704) | RAR vs RDW | <0.001 |
| RDW | 0.565 (0.521–0.609) | RAR vs Albumin | 0.014 |
| Albumin | 0.686 (0.644–0.727) | RDW vs Albumin | <0.001 |
| **XMHH cohort** |  |  |  |
| RAR | 0.715 (0.623–0.806) | RAR vs RDW | 0.092 |
| RDW | 0.643 (0.554–0.731) | RAR vs Albumin | 0.136 |
| Albumin | 0.663 (0.566–0.760) | RDW vs Albumin | 0.769 |

Table S8. Multivariable logistic regression analysis of RAR and Model 3 covariates for pneumonia

| **Variable** | **OR (95% CI)** | **P** |
| --- | --- | --- |
| RAR | 1.11 (1.02–1.21) | 0.019 |
| Age | 0.99 (0.98–1.01) | 0.325 |
| Male sex | 1.25 (0.89–1.77) | 0.202 |
| Heart rate | 1.02 (1.01–1.03) | <0.001 |
| Mean blood pressure | 0.99 (0.99–1.00) | 0.187 |
| Respiratory rate | 1.03 (1.00–1.06) | 0.054 |
| White blood cell count | 1.02 (1.00–1.04) | 0.042 |
| Glasgow Coma Scale | 1.00 (0.95–1.06) | 0.908 |
| Creatinine | 0.96 (0.85–1.07) | 0.444 |
| Blood urea nitrogen | 1.01 (1.00–1.02) | 0.026 |
| Alanine aminotransferase | 1.001 (1.000–1.002) | 0.027 |
| Aspartate aminotransferase | 1.000 (0.999–1.000) | 0.277 |
| Glucose | 1.001 (0.999–1.002) | 0.380 |
| rtPA use | 1.27 (0.69–2.31) | 0.442 |

Figure S1 Missing data visualization for MIMIC-IV and XMHH Cohorts. (A) MIMIC-IV cohort; (B) XMHH cohort.

Figure S2. ROC curves comparing RAR, RDW alone, and albumin alone for pneumonia discrimination in the MIMIC-IV and XMHH cohorts. (A) MIMIC-IV cohort; (B) XMHH cohort.

Figure S3 Bias plot of confounding relative risks for MIMIC-IV and XMHH Cohorts. (A) MIMIC-IV cohort; (B) XMHH cohort.

Figure S4. Nomogram for predicting pneumonia. (A) Nomogram based on age, RAR, heart rate, respiratory rate, WBC, BUN, and ALT. (B) ROC curve of the nomogram in the MIMIC-IV cohort. (C) Bootstrap-corrected calibration curve comparing predicted and observed probabilities. (D) Decision curve analysis of the nomogram.
